# The ultimate challenge to climate change: Endurance of a thermophilic reptile to the harsh temperatures on an extremely hot island

**DOI:** 10.1371/journal.pone.0320796

**Published:** 2025-04-30

**Authors:** Melissa Plasman, Aníbal H. Díaz de la Vega-Pérez, Marshall D. McCue, Mauricio Tepos-Ramírez, Víctor Hugo Reynoso

**Affiliations:** 1 Centro Tlaxcala de Biología de la Conducta, Universidad Autónoma de Tlaxcala, Tlaxcala, México; 2 Universidad Rosario Castellanos, Ciudad de México, México; 3 Consejo Nacional de Humanidades, Ciencias,y Tecnologías-Centro Tlaxcala de Biología de la Conducta, Universidad Autónoma de Tlaxcala, Tlaxcala, México; 4 Sable Systems International, Las Vegas, Nevada, United States of America; 5 Departamento de Zoología, Instituto de Biología/Pabellón Nacional de la Biodiversidad, Universidad Nacional Autónoma de México, Ciudad de México, México; HUN-REN Centre for Ecological Research, HUNGARY

## Abstract

Herbivorous ectotherms are especially vulnerable to climate change and those inhabiting hot environments may already live near their maximum physiological limits. Insular species are particularly susceptible to changing thermal conditions because they cannot relocate. This proves a very poor prognostic for the survival of herbivorous reptiles living on islands. The piebald chuckwalla, *Sauromalus varius*, is a large iguana endemic to San Esteban Island, located in the Gulf of California, encompassed by the Sonoran Desert, one of the hottest areas on earth. We investigated the thermal ecology of this iguana during the hottest month of the year coinciding with the fruiting of its most important food source, the giant cardon. We measured field body temperature (Tb_field_), voluntary maximum body temperature, the onset of thermal stress responses, and critical maximum temperature, and compared these with the thermal landscape. We found that Tb_field_ was 37.2±1.3°C (average±SD) and iguanas sought shade at a body temperature of 39.2±1.4°C. Iguanas started panting at 42.4±2.0°C, a cooling strategy at the expense of precious body water, and often defecated, at 43.2±1.9°C, with concomitant loss of water. We determined that these iguanas can maintain activity at body temperatures of 47.2±2.2°C, however they use various mechanical and behavioral mechanism to avoid these extremes. On the island, ground temperatures reached up to 62.4°C. Shade of plants can provide thermal shelter during part of the day. However, even in some caves temperatures could reach 41.5°C and under rocks 48.0°C, which is higher than these animals voluntarily tolerate. Our results indicated that although these chuckwallas can support high temperatures, their strategy incurs substantial water loss, a resource only available for the iguana through cacti consumption. Environmental temperature that increases with climate change will likely lead to an ever-increasing use of shelters, perhaps even resulting in complete inactivity during the cacti fruiting period.

## Introduction

Anthropogenic climate change is causing a sixth mass extinction event [1,2]. Among vertebrates, ectotherms are especially vulnerable to warming given their dependence on environmental temperatures for thermoregulation [3]. A recent correlative study found that herbivores are among the most vulnerable species, with the highest proportion of threatened species among herbivorous reptiles, with 52% of the species currently at risk [4]. The vulnerability of a species to climate change will depend on exposure, resilience to disturbance and potential for adaptation and, if all else fails, its ability to relocate to more suitable areas [3,5]. It is generally believed that climate change is too rapid to allow for adaptation [6]. Variation in thermal traits may allow for rapid adaptation, however at the warm end of the thermal tolerance range slow rates of evolution are mostly observed [7]. Hence, the survival of most species more likely involves changes in the distribution area. Distribution shifts are predicted and already observed among many lizards, causing local extinctions [8]. For terrestrial insular species the dispersal capacity is particularly restricted when large areas of water form important barriers [9] and their survival will depend on their capacity to tolerate warmer environments. Many desert dwelling species already live near their upper thermal limit [10,11] and increases in environmental temperatures may push them over their thermal threshold. This may especially be the case for desert dwelling reptiles [12,13].

Rising ambient temperatures can shorten the periods that allow activity, and extreme weather events can lead to lethal temperatures [14,15,16]. Nevertheless, studies rarely appreciate the thermal exposure of the individuals on a microhabitat scale [17,18,19]. This may be especially important in deserts where substrate temperatures can be much higher than the mean air temperatures (e.g.,[20]) that are normally included in climate change studies. Exposure to environmental temperatures depend on habitat structure and micro-habitat use by the animals [19,21]. For example, it was observed that bird species suffered more from climate change than burrow dwelling mammals in the Mojave Desert [22]. Shifts in behavior of species with burrows towards higher refuge use may reduce climate change impact and increase survival [23]. However, increase in use of shelters reduces activity, which may impose costs, like loss of foraging time [24].

The Sonoran Desert is one of the hottest places on earth [25]. Additionally, precipitation is scarce [26] and fresh water is mostly absent [27]. Even so, biodiversity is high, bearing a high number of endemics, including many herbivorous reptiles. Among these, chuckwallas (*Sauromalus*; Family: Iguanidae) are strict herbivorous reptiles [28] that inhabit several islands in the Gulf of California and the mainland. As non-swimmers, their dispersal capacity is very limited which has resulted in a high insular species diversification [27].

San Esteban Island, due to its concave shape, is notorious for its extreme high temperatures during the summer months. The chuckwalla of San Esteban Island, *Sauromalus varius*, is of special interest, as this species is active during the hottest period of the year coinciding with the ephemeral availability of their favorite (and ostensibly the most energetically important) food [29], the pitaya, the fruit of the giant cardon *Pachycereus pringlei*. During the months of July and August fruits fall to the ground and within reach of the iguanas [20].

Ectotherm species currently living in high temperature conditions may be more susceptible to global warming, because they may have body temperatures close to their physiological limits [12,30,31]. Understanding the thermal ecology of reptiles living in extreme warm conditions, may help to unravel the possible reach of physiological traits at high temperatures in this taxonomic group. Limits in physiological plasticity may already have been reached, restricting possible adaptation to even hotter climates during anthropogenic climate change [32]. Here, we investigated the field thermal ecology of the desert dwelling reptile, *Sauromalus varius*, on the San Esteban Island during the hottest period of the year and compare this with environmental temperatures to which it is exposed in different micro-habitats and shelters.

## Materials and methods

*Sauromalus varius* is listed in IUCN red list as vulnerable and is in the official Mexican Standard (Norma Oficial Mexicana [33]) categorized as endemic species for Mexico with risk factor “Threatened”. Individuals of *Sauromalus varius* were temporarily captured and handled under the permits of Subsecretaría de Política Ambiental y Recursos Naturales, number: SPARN/DGVS/06199/23. San Esteban Island is a protected natural area with restricted access, here permitted by Comisión Nacional de Áreas Naturales Protegidas (CONANP) oficio: APFF.IGC-Sonora/153/2023 and Secretaría de Gobierno, oficio: DAJCS/211/974/2023. Permits did not allow for extraction of iguanas from the island, for which tests were performed *in situ*, under natural conditions (i.e., without access to electricity and standardized laboratory conditions). Ethics on handling and experimental procedures were also evaluated and authorized in the permits SPARN/DGVS/06199/23 (13th of June, 2023) and APFF.IGC-Sonora/153/2023 (7th of Julio, 2023).

### Capture and maintenance of iguanas

The hottest months in the Gulf of California are July and August (temperature data for the states of Sonora and Baja California Sur, Mexico; [34]). Fieldwork took place in this period, between 17 and 24th of July 2023. Iguanas were captured by hand or with a noose and placed in a cloth bag. Iguanas were brought to the campsite on the island and near the capture site (< 1 km), where they were kept in the shade within a cave at temperatures between 30 and 36°C when not tested. We measured body size by snout vent length (SVL) with measuring tape (± 1 mm) and body mass (± 0.01 g; Radwag, WTB 3000). We determined sex of the individuals by inserting a sexing probe posterolaterally into the cloaca, trying to find the hemipeneal duct in males. In females, that does not have hemipeneal duct, the probe does not enter more that 15 mm. Iguanas were released at the site of capture within six days of capture.

### Thermal traits

In ectotherm species, body temperature is a primary determinant of physiological performance. In natural conditions, ectothermic species are expected to thermoregulate within the range of optimal body temperatures, because at temperatures above or below this range (suboptimal or pejus temperatures) fitness may quickly decrease [35]. To determine the range and limits of optimal body temperatures, we measured field body temperature and voluntary thermal maximum.

Field body temperature (Tb_field_): Body temperature was measured immediately after capture with a quick reading thermometer (Fluke 52 II; ± 0.1°C), by inserting a thermocouple (type-K) 3 cm into the cloaca. In daytime, *S. varius* are normally resting under the shade of pitaya cactus (*Stenoceros gummosus*), elephant trees (*Bursera microphyla*), and cholla cactus (*Cylindropuntia alcahes*).

**Fig 1:**
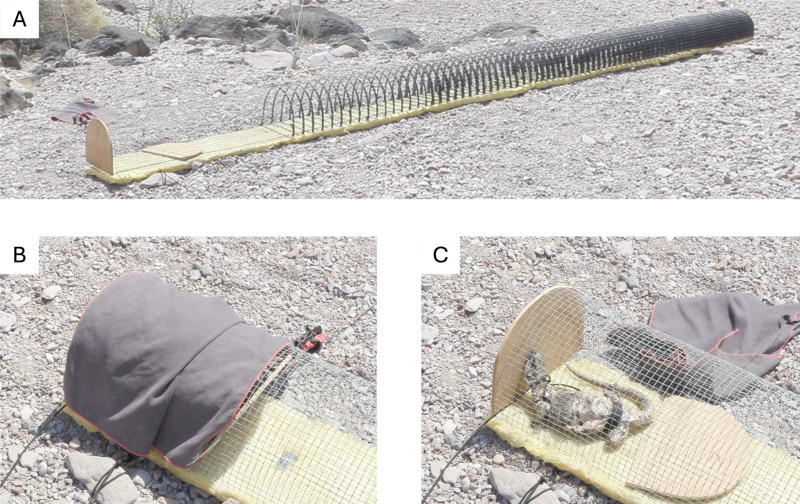
Experimental test to determine voluntary thermal maximum. **A)** Photograph of the cage used for the test. Size was 30 x 30 x 600 cm. Black ribbon braided through the mesh provided different shadings; 1) first meter without shade, then of every 5 cm (2) 1 cm was shaded (3) 2 cm shaded (4) 3 cm shaded, (5) 4 cm shaded, and (6) completely shaded. The cage was placed over fiberglass for thermal isolation from heat of the ground. **B)** The iguana was placed in the cage with a cloth covering it to reduce stress. **C)** After the researcher was out of sight, the cloth was removed, and the iguana could move freely throughout the cage.

Voluntary thermal maximum (VTM): We determined the body temperature at which the iguana sought shade. We designed a novel experimental set-up consisting of a 0.5 inch wire mesh cage 30 cm wide and high by 6 m long in which the iguana could freely move between full sun exposure and six sections of increasing shade intensity (Fig 1). Shade was made by braiding black ribbon through the mesh. The first meter did not have shading and was fully exposed to the sun, the second meter had shading of 1 cm every 5 cm, and every meter 1 cm of shading per 5 cm was added, until the last meter that was fully shaded. To prevent the iguana from over heating from the ground substrate, the cage was thermally isolated by placing it over a thick mat of fiberglass (2“) with an aluminum foil undersurface.

The iguana was initially placed in the non-shaded section of the device but was at first covered with a cloth to keep it calm. At the beginning of each recording, the researcher located at a hidden, distant position used a cord to remove the cloth, thereby fully exposing the iguana to the sun. As soon as the iguana warmed and moved away from the sunny area into shade, it was immediately extracted using a string previously attached around its chest and body temperature was measured with a thermometer (Fluke 52 II) 3 cm deep in the cloaca and recorded the shaded area into which it took refuge. We recorded body temperature immediately before the trial (Tb_initial_) and the time the iguana stayed in the sun before seeking refuge (Time_VTM_). Few trials in which iguanas ran into the shade because of external factors (e.g., nearby animal or startling noise), were discarded and tests were repeated the next day. Tests were performed 3.8±1.8 (average ± standard deviation) days after capture.

### Thermal tolerance

**Fig 2:**
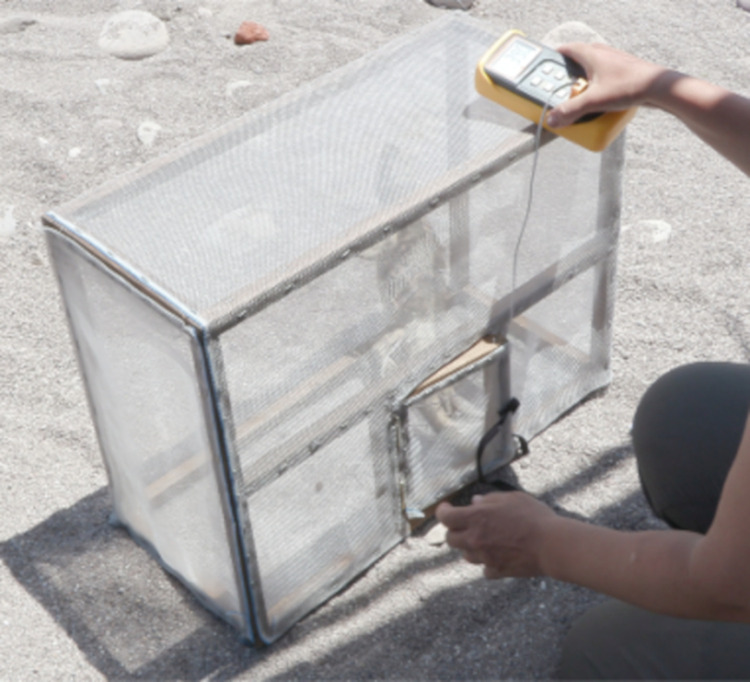
Experimental test to determine critical thermal maximum. The test consisted of a rectangular parallelopiped cage (60 x 40 x 25 cm) of a wooden frame covered with plastic mesh, which was placed in the sun. A thermocouple of the quick reading thermometer was inserted into the cloaca of the iguana for continuous recording of body temperature. The cage was tumbled every 30 seg.

To evaluate the upper thermal limit of physiological tolerance, we determined maximum critical temperature (CT_max_) as the thermal limit of locomotor performance. The iguana was placed in a rectangular parallelopiped cage of 60 x 40 x 25 cm made of a wooden frame covered with plastic mesh (Fig 2). A thermocouple of the quick reading thermometer was inserted into the cloaca and maintained there by taping the cord to the tail. As the experiment was designed to measure variables in natural conditions, the cage was placed in direct sunlight. This test is normally performed in standard laboratory conditions with an incandescent light as heat source [36].

Every 30 seconds body temperature was recorded, then the cage was turned over and the response of the iguana was registered. CT_max_ was considered the highest temperature at which the iguana could successfully turn over, before loss of locomotor capacity (i.e., not recovering from flipping and remain laying still with its belly up) the next time the cage was tumbled. We also recorded other behavioral responses of the iguana: panting (opening the mouth for cooling through evaporation of saliva), quick head movements (perhaps the onset of spasms), and spontaneous defecation. When the iguana defecated, the feces were quickly removed from the cage, and the test continued. When the iguanas collapsed and did not turn over after flipping, they were rapidly placed in the shade (at approximately 36°C) to cool down. When iguanas were still able to turn over after 50 min in the trial, we stopped the test and considered CT_max_ to be inconclusive, and we placed the iguanas in the shade to cool. We could determine CT_max_ in 10 iguanas. In the other five iguanas, CT_max_ was not reached most likely because environmental temperatures were not sufficiently high. Because of the high levels of stress and exertion during this test individual iguanas did not repeat the test. Tests were performed 2.5±1.7 days after capture.

### Thermal landscape

We determined thermal landscape by recording environmental temperatures in microhabitats available to the iguanas in their natural habitat (S1 Fig). We placed 32 temperature dataloggers (HOBO), that registered the temperature every 30 min during six of the hottest days of the year (18th to 23rd of July 2023). Dataloggers were placed in a range of different microhabitats with different levels of shading and possible shelters: three in the sun at 30 cm above the substrate, seven under bushes without leaves, six below bushes with leaves, five under rocks and four in caves. We also placed two dataloggers in the soil in a sunny area and two dataloggers in the soil in a shaded area; the sensor of the thermometer was placed 2 cm below the surface. Additionally, two dataloggers were placed at 150 cm above ground to measure average air temperatures on the island. Prior to the running period, dataloggers were tested and found to record very similar temperatures (coefficient of variation = 0.012±0.004). Dataloggers were used to measured environmental temperatures used for thermoregulation by the iguanas, and do not pretend to be a direct proxy for body temperature. Dataloggers do not have the thermal properties of the iguanas (mass, color, material, blood circulation, evaporation, etc.).

### Statistical analyses

We used general linear models (GLM) to determine the effect of sex and body mass on the thermal traits (i.e., CT_max_, panting, VTM, Tb_field_) and General linear mixed models (GLMM), to test for the effect of panting on the heating rate, comparing the heating rate over 10 min before and after the iguana opened its mouth. GLMM included individual identification as random factor and time (before or after opening the mouth), body mass, and sex as predictors. We tested for a parametric distribution using the Shapiro test over the residuals of the models; in all tests, residuals displayed a parametric distribution. Outliers were determined by visualization in box plots. Analyses were done in R, version 4.2.0 [37] using the package car [38] and lme4 [39].

We determined activity-time restriction (restriction hours) for each of the micro-habitats (i.e., sun exposed substrate, shaded substrate, under bushes with or without leaves, under rocks, and in caves), when their temperatures are above those tolerated by the iguana impeding activity of the animals. We considered restriction hours the time when temperatures in the micro-habitats were above the average of CT_max_, which would inhibit all activity, but also when temperatures in the micro-habitats exceeded temperatures of the onset of thermal stress responses (i.e., panting, VTM).

## Results

We obtained data from 15 adult iguanas (9 females and 6 males). Body size of experimental individuals was generally similar (Table 1) and did not differ between males and females for SVL (T-test: t_12.96_= -1.79, P = 0.11) and body mass (T-test: t_8.58_ = -1.81, P = 0.11).

**Table 1:** Morphological data and thermal traits of *Sauromalus varius* males and females.

	Males	Females
**Body size**		
SVL (mm)	305±69 (295-314) n=6	297±10 (284-315) n=9
Body mass (g)	985±117 (833-1151) n=6	885±87 (758-1009) n=9
**Thermal traits (°C)**		
Tb_field_	36.4±0.91 (35.2-37.5) n=5	37.2±1.0 (36.1-39.0) n=7
VTM	38.7±1.5 (36.5-40.8) n=6	39.5±1.2 (38.2-41.9) n=9
CT_max_	46.8±1.2 (45.0-47.5) n=5	47.5±2.9 (44.3-52.0) n=6
**Thermal stress (°C)**		
Panting	41.4±1.6 (38.9-43.5) n=6	42.4±1.1 (41.2-44.6) n=9
Defecation	42.1±2.1 (39.7-44.9) n=5	43.7±1.6 (42.0-46.4) n=5
Head spasms	45.2±1.2 (44.5-47.3) n=5	44.5±0.9 (43.9-45.9) n=4

Mean ± SD are given. Ranges are presented in parenthesis

### Field body temperature

The Tb_field_ was 36.9 ± 1.0°C (Table 1). Males and females did not differ (F_1,7_ = 1.91, P = 0.21) and Tb_field_ was not affected by body mass (F_1,7_ =1.02, P = 0.35). The same model showed that Tb_field_ was not correlated with substrate temperature (35.1 ± 1.5°C; F_1,7_ = 1.21, P = 0.31) or air temperature (34.4 ± 1.5°C; F_1,7_ = 1.90, P = 0.21) at the location of capture.

### Voluntary maximum body temperature

Even though iguanas had access to different shading in the test, they always went into the fully shaded area when leaving the section exposed to the sun. VTM was 39.2 ± 1.4°C (Table 1). VTM was not affected by sex (F_1,10_ = 0.24, P = 0.63), temperature within the gradient (F_1,10_ = 1.34, P = 0.27) or body mass (F_1,10_ = 0.81, P = 0.40). VTM was positively correlated with initial body temperature at the start of the trial (F_1,10_ = 5.61, P = 0.04; estimate = 0.90 ± 0.38 SE). Unexpectedly, the time for the animal to reach its VTM was not affected by the temperature in the gradient (F_1,10_ = 0.39, P = 0.55); when the variables initial body temperature (Tb_inicial_: F_1,10_ = 1.06, P = 0.33) and VTM (F_1,10_ = 0.71, P = 0.42) were also included in the model.

### Maximum critical thermal limit

The CT_max_ averaged 47.2 ± 2.3°C (Table 1). Even iguanas that did not reach the point at which the righting response was lost, exhibited body temperatures as high as 45.8 ± 1.1°C (however these individuals were excluded from the analyses). The CT_max_ did not differ between the sexes (F_1,7_ = 0.76, P = 0.41) and did not relate with body mass (F_1,7_ = 0.86, P = 0.38). Postural or physiological thermoregulation efforts may have kept the iguanas at below critical temperatures for some time, but not indefinitely. For example, one of the iguanas maintained the body temperature stable at 45.7°C over 7 min, but then its temperature suddenly increased by almost 2°C in 30 sec. Thermoregulation efforts observed during the trials were assuming a position lateral to the radiation of the sun or assuming a vertical position with the lighter colored belly towards the sun, possibly to reduce radiation absorption, and, most critically, panting (i.e., opening the mouth for evaporative cooling). These behaviors were observed in all trials.

Panting was observed in all trials. The onset of panting occurred at a body temperature of 42.0 ± 1.4°C (Table 1). This temperature did not depend on sex (F_1,12_ = 3.6, P = 0.08) or body mass (F_1,12_ = 1.86, P = 0.20), and was not correlated with CT_max_ (F_1,8_ = 0.30, P = 0.59). Heating rate was not changed by panting (GLMM: F_1,23_ = 0.56).

During the CT_max_ trials, 10 iguanas defecated, which is ostensibly a reaction to thermal stress. Defecation occurred at an average temperature of 42.9 ± 2.0°C (Table 1). These temperatures were not correlated with CT_max_ (F_1,4_ = 0.02, P = 0.90), however they were positively correlated with temperature at the onset of panting (estimate = 1.08 ± 0.17 SE; F_1,8_ = 39.55, P = 0.0002). The temperature of defecation was not related to body mass (F_1,7_ = 3.39, P = 0.11), or sex (F_1,7_ = 4.91, P = 0.06).

During the CT_max_ trials, iguanas typically exhibited repetitive arrhythmic head movements, observed in 9 (out of 15) iguanas (4 females and 5 males) at 44.9 ± 1.1°C. The temperature at which these first occurred did not relate to CT_max_ (F_1,4_ = 0.08, P = 0.79), but sample size was small (n = 6). The onset of arrhythmic head movements was negatively associated with temperature of panting (estimate = -0.74 ± 0.27; F_1,7_ = 7.3, P = 0.03). This statistical relation was caused by one iguana that moved its head at high temperatures but had opened its mouth at low temperature. Excluding that case, the relation was not significant (F_1,6_ = 0.0007, P = 0.98).

During the trial, body temperature increased at a rate of with 0.20 ± 0.06 °C per min. Heating rate decreased with higher initial body temperatures (F_1,10_ = 6.25, P = 0.03; estimate = 0.017 ± 0.007), but was independent from body mass (F_1,10_ = 0.58, P = 0.46) and sex (F_1, 10_ = 0.06, P = 0.81). The time it took for iguanas to initiate panting was 17 ± 9 min, and to reach CT_max_ was 46 ± 8 min. Unexpectedly, time to CT_max_ was neither correlated with nearby air temperature at 150 cm above the ground (F_1,6_ = 0.05, P = 0.83) nor the temperatures of the substrate (F_1,7_ = 0.002, P = 0.96), when corrected for the CT_max_ (F_1,7_ = 2.12, P = 0.20). The time required to initiate panting was positively correlated with the substrate temperature (obtained from the nearby dataloggers; F_1,11_ = 11.99, P = 0.005), but not air temperature (F_1,7_ = 3.54, P = 0.09), and was positively correlated with the temperature at the onset of panting (estimate = 3.47 ± 1.19, F_1,7_ = 8.85, P = 0.01).

### Thermal landscape

**Fig 3:**
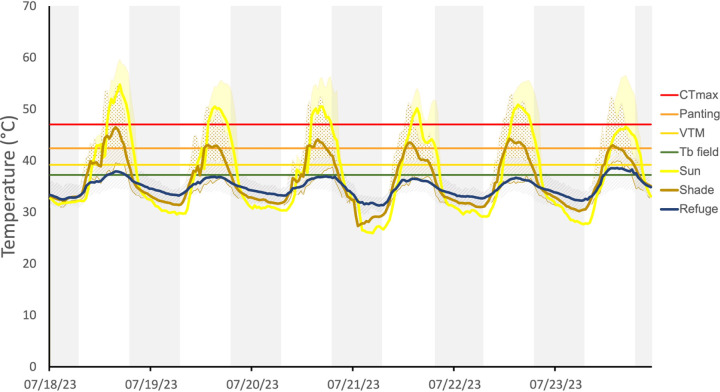
Environmental temperatures on San Esteban Island. Temperatures in the different micro-habitats (sun, shade, and shelters) during the hottest month of the year are shown; line of average and shaded area for the complete range recorded. Horizontal lines indicate the averages of the thermal traits of *Sauromalus varius*. Areas shaded in grey indicate nighttime.

Detailed thermal landscape data can be found in the database [40] and is summarized in Table 2 and Fig 3. During the daytime, differences in temperatures among micro-habitats were large, especially at the hottest time of the day. Whereas maximum air temperature at a height of 150 cm averaged 40.0±1.6°C (range 38.3–42.5°C), at a height of 30 cm this increased to 50.9 ± 5.0°C (range 41.1–59.7°C) and maximum substrate temperatures reached 55.4 ± 5.6°C, with smothering temperatures of up to 62.4°C. Vegetation offered considerable thermal buffering. Although the average decrease between substrate in the shade and that exposed to the sun was only -3.2 ± 5.4°C (during the daytime), at some times the difference was as much as 18.6°C. Compared to substrate in the sun, temperatures below bushes dropped an average of 5.3°C, but could differ with more than 25°C during the hottest hours of the day, when substrate temperatures were above 60°C. Below rocks, temperature was on average 7.9 ± 7.4°C lower than substrate temperature in the sun, but during the hottest hours differences could reach to 25.6°C. In deeper shelters (i.e., caves) temperature averaged 10.1 ± 8.0°C lower than sun exposed substrate temperatures and reaching differences of 27.7°C.

**Table 2:** Thermal landscape available to *Sauromalus varius* on the San Esteban Island, Mexico.

Daily temperature measures	Air (150 cm above the ground)	Sun (30 cm above the ground)	Substrate in the sun	Substrate in the shade	Below plant without leaves	Below plant with leaves	Below rocks	In caves
**Temperatures (°C)**								
Daily average	34.3 ± 0.9	37.0 ± 2.1	40.2 ± 2.1	38.3 ± 1.6	36.1 ± 1.3	36.0 ± 0.9	34.7 ± 2.5	33.6 ± 0.8
Daily minimum	30.6 ± 1.4	28.8 ± 1.9	32.1 ± 2.0	33.6 ± 1.9	30.5 ± 1.7	30.4 ± 1.7	30.8 ± 4.0	31.9 ± 1.3
Daily maximum	40.0 ± 1.6	50.9 ± 5.0	55.4 ± 5.6	44.5 ± 3.0	45.9 ± 4.8	45.1 ± 3.4	40.1 ± 2.7	35.9 ± 1.9
Highest temperature recorded	42.5	59.7	62.4	51.4	54.1	54.5	48.0	41.5
**Restriction hours (h) considering:**								
CT_max_	0.0 ± 0.0	3.6 ± 2.9	4.8 ± 2.3	0.1 ± 0.3	1.0 ± 1.4	0.4 ± 0.9	0.0 ± 0.2	0.0 ± 0.0
Panting	0.0 ± 0.1	5.3 ± 3.1	6.2 ± 3.2	3.0 ± 3.4	3.0 ± 2.6	2.7 ± 2.3	0.3 ± 0.8	0.0 ± 0.0
VTM	1.1 ± 1.9	8.5 ± 1.5	10.0 ± 1.7	9.6 ± 3.2	6.5 ± 2.6	7.0 ± 1.0	3.4 ± 3.4	0.3 ± 1.6

Daily temperature measures were obtained with dataloggers placed in micro-habitats that registered every 30 min during 6 days in July 2023.

### Restriction in activity

**Fig 4:**
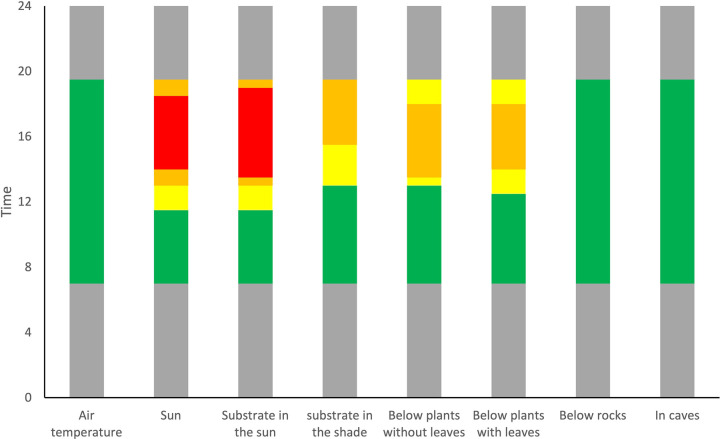
Restriction in activity during the summer of *Sauromalus varius.* Restriction in activity of the iguana in its natural habitat is shown in red when environmental temperatures are above CTmax, orange above the gaping threshold, and in yellow when above VTM. Green indicates time when temperatures are below VTM and grey indicates nighttime. Environmental temperatures were averaged per microhabitat for all days of measurement.

When considering the limit of thermal tolerance (CT_max_), in sun exposed areas activity was restricted for 4.8 ± 2.3 h per day. However, restricted time quickly reduced in shaded areas (Table 1), where (except for below one open bush without leaves) restriction hours were less than 2 h and often 0 h. Both rocks and caves provided adequate thermal shelter without restriction hours. However, if these iguanas are to avoid thermal stress, restriction hours increased (Table 1). On the hotter days, sun exposed substrate reached temperatures above the panting threshold during 9.5 h per day. In plant shaded areas restriction hours ranged from 0 h to a maximum of 7 h. Below one rock 3.5 h and another two rocks 1 h of restriction was found, whereas the other three rocks and all caves provided adequate thermal shelter during the entire day. Nevertheless, it appears the iguanas restrict themselves further. Considering VTM, restriction hours could reach a maximum of 12 h, of the 12h 30 min of daylight during the period of measurements, in sun exposed substrate and even substrate in the shade (Table 1; Figs 3,4). Below vegetation restriction hours could range from 0h to 9.5 h, although this depended on the day and bush. Interestingly, below some rocks, temperatures could be above VTM for 8.5 h on some days. Temperatures in the caves did not reach above VTM, with the exception of one cave that had 8 h of restriction during the last day of measurements.

## Discussion

Here we studied the thermal ecology of an extreme thermophile vertebrate, *Sauromalus varius* and its thermal landscape, during the hottest month of the year in its severe hot environment. We found that this desert iguana can endure high temperatures before losing locomotor capability; however, they prefer to seek shelter at much lower temperatures. We also found signs of heat stress at temperatures much lower than CT_max_, which suggest a decline in fitness well before reaching the maximum temperature they can actively tolerate. Conservation efforts will be especially important for insular populations or species that cannot alter their geographic distribution. To perform timely conservation actions as a response to anthropogenic climate change, we should evaluate the detrimental effect of warming and determine when temperatures exceed critical thermal limits. Characterizing the thermal thresholds of a species directly in natural conditions, is particularly valuable because animals will not go through the stress of transportation and acclimatization to laboratory conditions before experiments that may modify their natural responses to heat.

### Heat stress: fundamental limits vs realized thermal limits

The CT_max_ of *S. varius* was as high as 47.2°C, higher than that reported for *Sauromalus ater* in Riverside (46.6°C [41]) and the Mohave Desert (46.7°C [42]), in California, USA. This is considered extreme given that only 3.5% of studied squamate reptiles have CT_max_ above 47°C [43]. Interestingly, one individual reached a body temperature of 52°C before being unable to flip over, which is higher than reported for any other *Sauromalus* species, and even above the lethal temperature of *S. obesus* (50–51°C [42]). The highest reported temperature for other reptile species is 52.4°C of *Aspidoscelis sexlineatus* [44]. It should be noted that Paulissen defined CT_max_ as the temperature of the onset of spasms which is expected to be higher than loss of locomotor capabilities and close to lethal temperature [45]. Since we decided not to measure temperature at muscle spasms or lethality in this threatened endemic species, we cannot make exact comparisons with that previous study.

In reptile studies, body temperature is typically measured within the cloaca, although body temperature may differ among body parts. For example, temperature may differ between the cloaca and the brain, where increases in brain temperature may be much more critical than in other parts of the body [46]. However, in large lizards [46] and iguanas [47] temperature did not differ much between the brain and cloaca.

Here, CT_max_ was tested under natural conditions using the loss of righting response, the most used technique in literature [36]. In this tests, fatigue of flipping may occur [36,48], although we cannot exclude a possible effect of fatigue, without the frequent confirmations of the rightening response our estimates of CT_max_ would likely be even higher. In any case our results suggest that *S. varius* may be the vertebrate species most tolerant to extreme heat studied so far.

Air temperatures measured in the same month as the thermal traits, at 150 cm above the ground, reached up to 42.5°C, well below CT_max_, however air temperatures in the sun at 30 cm above the substrate temperatures approached 60°C and substrate temperatures exceeded 62°C (Table 1). It has been reported that substrate temperatures on the island will occasionally reach 70°C in the sun [20]. *Sauromalus* are crawling reptiles that rarely climb bushes or trees [27; *Pers. Obs.*], and these high substrate temperatures are probably the most important limiting factor for activity restriction.

Our results indicate that at least in the summer months these iguanas are subjected to almost 5 h of restriction in the middle of the day, in which the iguanas cannot stay on the sun-exposed substrate. However, they can still rest in shaded or partially shaded areas, like under bushes, for almost the entire day without reaching critical temperatures. Accordingly, all iguanas were found in shaded areas during this study.

Importantly, we observed evidence of heat stress at much lower temperatures than CT_max_. Iguanas started panting at 42°C, at a slightly lower temperature than other squamates living in the Sonoran Desert. Panting occurs around 42.5–43°C in *Crotaphytus collaris* and 43–44°C in *Dipsosaurus dorsalis* [45]. Evaporative cooling is little used in reptiles, although it can occur through the eyes and cloaca, and animals may actively induce evaporation by opening their mouth. In reptiles, panting may cool down the body to 5°C below air temperature [49]; however, mostly responses are much smaller [41]. In *S. ater* panting only allowed for a difference of 1.7°C between Tb and environmental temperature [41]. In this study, we did not find a noticeable decline in body temperature or in heating rate when iguanas opened their mouth. However, we measured body temperature in the cloaca and panting may have reduced temperature in the brain [46]. Evaporative heat loss in the absence of water may impose high costs of panting through dehydration and lowering survival. Dehydration impedes attainment of physiological homeostasis, and a good water balance is crucial for biochemical reactions, cell metabolism, and whole-body performance [50]. Dehydration can elevate panting threshold [51,52] and differences in state of hydration may explain the large range in temperatures at the onset of panting (38.9–48.1°C). A state of dehydration also typically lowers CT_max_ and reduces activity time in hot environments [53]. On San Esteban Island direct drinking water is absent, and rehydration must occur by extracting water from food. The lack of observation of panting in wild *S. varius* implies that the limited benefit of panting and the high additional survival costs makes this behavior undesirable, and panting is an indisputable sign of critical heat stress in desert animals [41].

Additionally, in 10 out of 15 trials iguanas defecated. Defecation, with its concomitant loss of water, is an unexpected response to thermal stress. Temperatures above the thermal tolerance range may halt the digestion [54] and foment the discharge to avoid fermentation within the digestion tract. However, the loss of water [50] and possible nutrient uptake may deteriorate the condition of the animal. Energy requirements increase with body temperature in ectotherms [55] and higher food intake will be needed, demanding more foraging time in the hotter times of the year when thermoregulation within the thermal tolerance range is more difficult.

We observed repetitive arrhythmic head movements in several iguanas which may be the onset of muscle spasms. Muscle spasms occur near lethal temperature in *Amphibolurus muricatus* (at 43°C, with lethal of 43.6°C [49]). These findings indicate that at temperatures below CT_max_ encompasses a stressful range of temperatures that will result in heat failure and death [56].

Furthermore, body temperatures maintained at temperatures at which performance is suboptimal (called pejus temperatures) may significantly reduce survival and increase local extinction rate [57]. This is especially true for species with a slow individual turn-over [57] and in *S. varius* reproduction rate is extremely low [27]. VTM is considered a good indicator of the upper limits of optimal temperatures [58]. VTM of *S. varius* was 39.2°C and much lower than CT_max_ (47°C). Hence, fundamental thermal limits may indicate fewer restriction hours than the realized thermal limits (*sensu* [11]).

Restriction based on CT_max_ reached up to 4.8 h (Table 1); however, based on VTM restriction increased to 10 h for temperatures measured in the substrate in the sun. Then, during the month with the highest abundance of quality food, foraging may be limited to just 2.5 h per day in sun exposed areas, or the iguanas may be restricted to shady areas where they may only have access to poor quality food. Nevertheless, the shade of the cactus itself, nearby bushes, and shelters can function as thermal shelters near the food source. The chuckwallas are observed below cardon cacti, circling with the shade movement, waiting for the fruit to drop. Then, only around noon, when shade is absent, shelter may need to be sought.

Restricted foraging limits energy intake, and therefore restricts body maintenance, growth, and reproduction [59]. In *Sceloporus* species restrictions hours of 3.85 h or more is predicted to result in local extinction [8], much lower than restriction hours found in this study, at least in sunny areas. Moreover, some apparent shelters (below rocks) also reached temperatures above VTM, suggesting that iguanas may have to tolerate pejus temperatures or take refuge in caves. Deep caves, extending several meters from the entrance, are present on the island, and the iguanas appear to progressively excavate them through the years (VHR pers. obs.).

### Time to reach heat failure: Ecological implications

The impact of a stress factor, like thermal extremes, depends on both intensity and duration [60,61]. We found that body temperature increase was quite slow. Because we tested the iguanas in natural conditions, body temperature increased at a lower rate (~0.2°C min^-1^) than in most laboratory studies (~1°C min^-1^ [36]). However, testing in natural conditions gives us a better idea of body temperature increase in free-ranging individuals. We found that in natural conditions iguanas could stay in the sun for 46 min before reaching thermal limits. However, thermal stress as indicated by panting was reached much earlier (18 min) and VTM in 7 min. During the summer when the chuckwallas favorite food is available, foraging could only take place in few hours of the day, or possibly in short periods with the risk of reaching above permissive body temperatures and enter in heat stress. Some reptile species are known to survive with short foraging times, like the turtle *Testudo horsfieldi* that had activity times of little over an hour and fed during 8–15 min bouts [62]. Although, short foraging times are usually associated with high food abundance and food quality (e.g., [63,64,65]), in deserts food availability is generally low and of low quality [66], and the effect of restriction hours may be more strongly pronounced.

In the absence of fruit of cacti, in the feces of *S. varius* several plant species could be visually identified (unpublished data), and the iguanas may eat from the plants within the shadow of the food source, avoiding the long restriction times in sun exposed areas. Although, considering the apparent lack of assimilation, the nutritional value of these plants may be low. Their large body size of these chuckwallas may buffer against low food assimilation (or foraging ability) in certain seasons [27]. However, summer is the season in which the favorite food source of *Sauromalus*, the pitaya (the fruit of the giant cardon) is available. With a high water and energy content, the pitaya has an exceptionally high nutritional value in the Sonoran Desert environment [29]. *Sauromalus* is not a good climber [27], so it highly depends on the feeding activity of the iguana *Ctenosaura conspicuosa* that also lives in San Esteban Island, which climbs the cardon to eat the pitayas, usually droping them while eating. During the hot summer months, the iguanas may seek refuge in thermal shelters near the giant cardon, until the fruits fall within reach, and perhaps only then risk exposure to high temperatures.

Increase in body temperature during the critical temperature trials may have been slowed by possible thermoregulation efforts by the iguanas. In the trials we observed the iguanas to orient their body lateral to the radiation of the sun. Body orientation can increase or decrease capture of solar radiation and thereby affect body temperature of ectotherms [49,67]. This effect can be further increased by skin color, as dark colors absorb more radiation and heat, and lighter colors may reflect more radiation reducing body temperature [68]. Some reptiles are even able to change color with body temperature to reduce time spent on thermoregulation [69]. *S. varius* did not exhibit changes in color, however they were observed to direct their belly towards the sun. The ventral side of *S. varius* is light colored and thus likely reflects more solar radiation than the dorsum (black, orange and yellow coloration). Further, during the trials, iguanas were observed to climb the mesh of the cage. This behavior may aid in thermoregulation as it moves them away from the hot substrate. Surface temperatures in sun exposed area was much hotter than air temperature, even a short distance from the substrate. We found that temperature could be more than 10°C lower at a height of 30 cm compared to the substrate temperature. Microhabitat selection is considered to have the strongest effect on body temperature regulation in lizards [70]. In the present trials, movement was limited and the iguanas could not prevent the increase in body temperature, although they may have slowed the rate of temperature increase. The above suggests that even in sun exposed areas, small postural adjustments can delay heat failure.

### Vulnerability to climate change

Climate change appears severe in the Sonoran Desert; extreme climate events have increased in number [13,71] and temperatures are predicted to increase rapidly [72]. Thermal requirements in *S. varius* were similar to those of other desert reptile species (see above), which may indicate that reptile species inhabiting high temperature environments are near their physiological limits [11]. This would mean little possibility for acclimation or adaptation to even hotter climates.

Vulnerability to climate change will depend largely on the thermal landscape structure, which in turn depends on sun exposure, vegetation, and shelter availability [21,73,74]. Our thermal data suggest that even now some shelters can reach temperatures above the voluntary body temperature range of *S. varius*, suggesting a loss of thermal shelters with increasing temperatures. Deep caves will be required for thermal shelters and their availability essential for survival. It will need to be evaluated if the deep caves present on the island are sufficient for future survival of the species. The temperature data obtained in caves suggest temperatures generally remain below VTM and will likely provide sufficient shelter against the increasing environmental temperatures. However, refuge use will restrict foraging. Realized thermal limits in sunny areas, where high quality food sources (pitayas) are likely most abundant, suggest large periods of restriction in foraging under current climate conditions. Increases in environmental temperatures as predicted (see [72]) would further increase the restriction hours in sun-exposed areas. Currently, shade provided by plants provide thermal shelter during most of the day, however climate warming may cause rises in temperatures within the shaded areas, as well. More importantly, with an increasing frequency of drought events in the Sonoran Desert [75] plant cover may reduce, increasing the area directly exposed to the sun and reducing the thermal shelters [73]. This would result in a more homogeneous thermal environment with higher mean temperatures, often far above the optimum temperature range, both factors reducing thermoregulation efficiency [76], leading to an ever-increasing hostile environment. Furthermore, if the iguanas are not able to eat the fruit of the giant cardon and protect the seeds within, because seed germination strongly reduces when seeds are exposed to the high surface temperatures, loss of this cacti on the island seems to be inevitable [20]. Although thermal shelters are available, *S. varius* is likely to lose its most nutritionally important food item due to thermal restrictions.

It is currently unclear when restriction hours will cause a collapse in population survival, as some reptile species are known to survive well with short foraging times [62]. In future conditions active temperatures may be restricted to the nighttime. Whether species will be able to adjust from diurnal to nocturnal foraging is currently unknown, and although energetically it may be feasible [77], other characteristics may be required. Iguanids are diurnal reptiles. Diurnal species generally present higher abundancy of cones than rods in the retina of the eyes. In some diurnal reptiles, rods, and hence night vision, are completely absent (e.g., [78,79]), making nighttime foraging difficult.

In this study, tests were performed during the hottest period of the year, as such here we showed the situation in the most extreme climate that this species of iguana currently experiences. In a future of climate change, these conditions of thermal stress observed in summer, can extend for a longer period. Currently, this iguana has survived managing a cold winter when it estivates for most of the time, and a summer where it may shelter for a large part of the hot days. This summer is an annual barrier that the iguana must survive to reach the reproduction period and after that the estivation. Even though pitayas are present every summer, in some years of extreme drought fruits do not mature. This means that in some summers the iguanas do not receive any benefits, and even so, they must survive the high temperatures. Extreme drought has already been associated with adult mortality (VHR, *pers. com*. in [80]). The predicted increase in these critical years [73], will be the determining factor in the survival of these iguanas.

San Esteban Island is a natural protected reserve, because of its unique biodiversity. The main objective is to permit the continuity of natural processes, under conditions of minimal human interference. Human activity is restricted to scientific investigation and conservation efforts. For the continuity in iguana survival on San Esteban Island, it is important to avoid habitat degradation and so maintain an adequate and heterogenous thermal landscape and food availability. Iguana and cardon (with its fruit formations) populations should be closely monitored.

## Conclusions

This desert dwelling reptile lives in an extreme hostile environment. Environmental temperatures were among the highest measured on earth [25]. We found that *S. varius* can endure high temperatures, albite at a high cost and they seek shelter at much lower temperatures. Short activity periods in sun exposed areas may be possible during the day in the summer months, but risk of heat stress and possibly death is high, and the iguanas are mostly restricted to shaded areas and refuges.

Additionally, it is very important to determine the local micro-habitat temperatures, as these can strongly differ from mean environmental air temperatures. The coarse thermal data generally used for the determination of thermal landscape mask real temperature exposure of the animals [81]. For *Sauromalus*, as a crawling species, substrate temperature is likely more important than air temperatures, and substrate temperatures reached much higher temperatures than air temperatures, especially in the sun.

Perhaps most importantly, our results show that it is important to distinguish between fundamental and realized thermal limits. Realized thermal limits likely much better represents survival probability of the species over longer periods of time in chronic warm environments. The difference between fundamental and realized restriction time was large, especially in micro-habitat that allows for foraging opportunities.

## Supporting information

S1 Figure Microhabitats of *Sauromalus varius* on San Esteban Island, Sonora, Mexico. Temperature dataloggers were placed in these and similar microhabitats. A) general view of the area. B) air temperature at 150 cm height. C) sun exposed area at 30 cm. D) substrate in the sun (in front) and shade (behind the rock). E) below a bush without leaves. F) below a bush with leaves. G & H below a rock. I & J) caves.
(TIF)
