# Supplementary figures and images for: The ultimate challenge to climate change: Endurance of a thermophilic reptile to the harsh temperatures on an extremely hot island
Source: PLoS One. 2025 Apr 30;20(4):e0320796. doi: 10.1371/journal.pone.0320796 (PMC12043117; doi:10.1371/journal.pone.0320796)

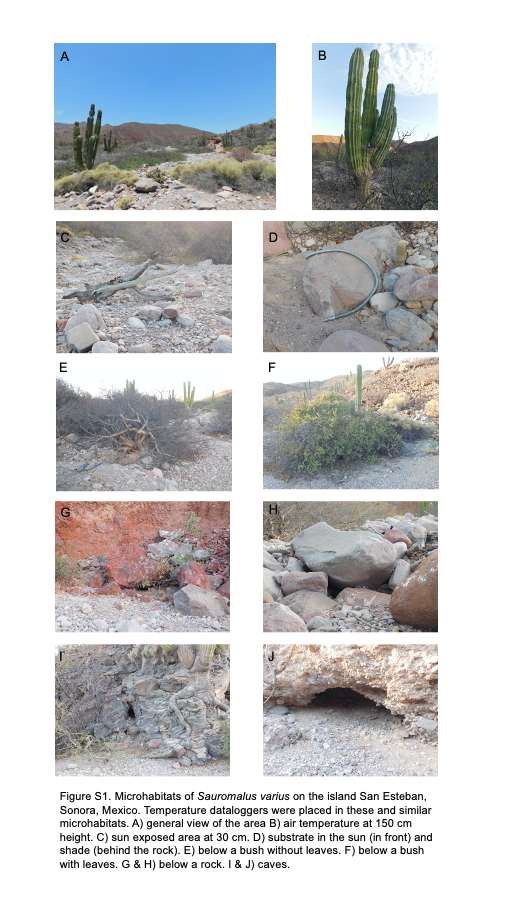

Supplement: S1 Figure — Temperature dataloggers were placed in these and similar microhabitats. A) general view of the area. B) air temperature at 150 cm height. C) sun exposed area at 30 cm. D) substrate in the sun (in front) and shade (behind the rock). E) below a bush without leaves. F) below a bush with leaves. G & H below a rock. I & J) caves. (TIF) [file pone.0320796.s001.tif]
